# An Atypical Presentation of Pediatric IgA Vasculitis: Early Hypertension and Severe Systemic Manifestations Despite Initially Normal Urinalysis

**DOI:** 10.7759/cureus.105716

**Published:** 2026-03-23

**Authors:** Samantha C Escobar, Mohammed A Alhendy, Debosree Vicsocsean

**Affiliations:** 1 Family Medicine, A.T. Still University School of Osteopathic Medicine in Arizona, Mesa, USA; 2 Pediatrics, Family HealthCare Network, Visalia, USA; 3 Epidemiology and Public Health, A.T. Still Research Institute, A.T. Still University School of Osteopathic Medicine in Arizona, Mesa, USA

**Keywords:** henoch-schönlein purpura, hypertension, iga vasculitis, leukocytoclastic vasculitis, palpable purpura, pediatrics, renal involvement, urinalysis

## Abstract

Immunoglobulin A vasculitis (IgAV), formerly Henoch-Schönlein purpura, is the most common systemic vasculitis of childhood and is typically self-limited. Renal risk stratification frequently relies on the presence of hematuria or proteinuria; however, early disease severity may not be detected by urinalysis alone.

We present a previously healthy seven-year-old boy with palpable purpura and joint pain. The diagnosis was made clinically based on the presence of nonthrombocytopenic palpable purpura in the setting of arthralgia. He was diagnosed with IgAV after initially reassuring laboratory findings, including normal renal function and repeatedly negative urinalysis. Despite this, his clinical course rapidly progressed to persistent vomiting, abdominal pain, gastrointestinal bleeding, scrotal edema, and sustained hypertension requiring hospitalization and pharmacologic management.

Repeated urinalyses during the acute illness remained negative for hematuria and proteinuria despite the development of hypertension and severe systemic involvement. This case highlights a clinical pitfall: although IgAV is primarily a clinical diagnosis based on characteristic findings such as palpable purpura and systemic symptoms, early laboratory studies, including urinalysis, may appear reassuring despite evolving systemic involvement. Close follow-up with serial blood pressure monitoring and urinalysis is essential during the disease course, whether initial laboratory findings are reassuring or abnormal.

## Introduction

Immunoglobulin A vasculitis (IgAV), formerly known as Henoch-Schönlein purpura, is the most common small-vessel vasculitis of childhood and is characterized by deposition of IgA-containing immune complexes within small vessels. The disease classically presents with palpable purpura, arthralgia, gastrointestinal involvement, and renal disease [1]. Although most pediatric cases follow a self-limited course, some patients develop significant systemic complications requiring hospitalization and close monitoring [2]. Renal involvement is the primary determinant of long-term morbidity, and screening typically relies on urinalysis to detect hematuria or proteinuria as early markers of glomerular inflammation [3]. Renal involvement, typically manifesting as hematuria and/or proteinuria, occurs in approximately 20-50% of pediatric patients with IgAV [4].

The underlying pathophysiology involves abnormal glycosylation of IgA1 molecules, producing galactose-deficient IgA1. These altered IgA1 molecules form circulating immune complexes that deposit in the small-vessel walls, triggering complement activation and leukocytoclastic inflammation [4]. Infectious triggers are frequently implicated in disease onset, particularly upper respiratory infections, although multiple bacterial and viral pathogens have been associated with IgAV [3,4]. While renal involvement often develops during the disease course, the severity of systemic manifestations may not correlate with early urinalysis findings.

The diagnosis of IgAV is supported by the EULAR/PRINTO/PRES classification criteria, which include palpable purpura with lower-limb predominance plus at least one of the following: diffuse abdominal pain, arthritis or arthralgia, renal involvement (hematuria and/or proteinuria), or histopathologic evidence of leukocytoclastic vasculitis with predominant IgA deposition [5].

We describe a pediatric patient with severe systemic manifestations and persistent hypertension despite repeatedly normal urinalysis, emphasizing limitations of early renal risk stratification and highlighting the importance of longitudinal clinical monitoring.

## Case presentation

**Figure 1:**
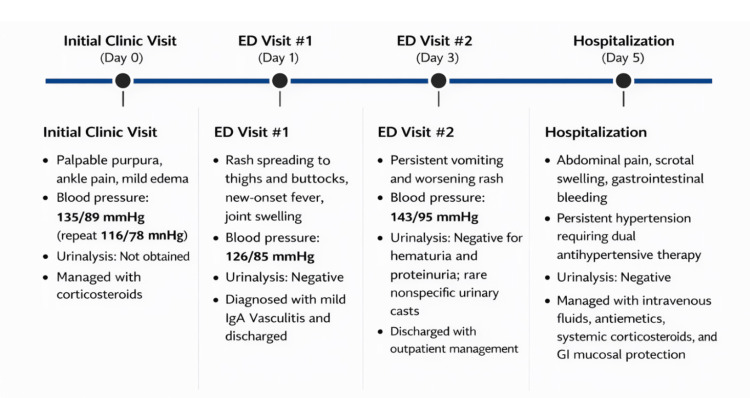
Clinical course of pediatric IgA vasculitis during the acute illness ED: emergency department; IgA: immunoglobulin A Graphical timeline illustrating the chronological progression of symptoms, vital signs, urinalysis findings, and clinical management across successive encounters, including the initial clinic visit, two emergency department visits, and subsequent hospitalization

A previously healthy seven-year-old boy with no significant past medical history presented to the outpatient clinic with a one-day history of right ankle pain and a nonpruritic rash over the lower extremities following minor blunt trauma. The rash was initially suspected to be allergic in etiology. He was treated symptomatically with ibuprofen 15 mL every eight hours, prednisolone 7.5 mg daily, and loratadine 5 mL daily for presumed allergic dermatitis. A right foot radiograph (three views) was obtained and was negative for fracture. No laboratory testing was performed at that time. This encounter represents Day 0 of the clinical timeline illustrated in Figure 1.

Within 24 hours of this initial clinic visit, the patient developed worsening extremity pain, fever, and extension of the purpuric rash to the thighs and buttocks. He was evaluated in the emergency department, where laboratory studies demonstrated normal platelet count, normal renal function, and urinalysis negative for hematuria and proteinuria. The clinical diagnosis was supported by fulfillment of the European Alliance of Associations for Rheumatology/Paediatric Rheumatology International Trials Organisation/Paediatric Rheumatology European Society (EULAR/PRINTO/PRES) classification criteria, including palpable purpura involving the lower extremities and buttocks, together with acute arthralgia manifested by ankle pain and impaired ambulation. The patient appeared clinically stable, was able to tolerate oral intake, and had no abdominal pain at that time. He was therefore diagnosed with IgAV and discharged home with outpatient management and follow-up.

**Figure 2:**
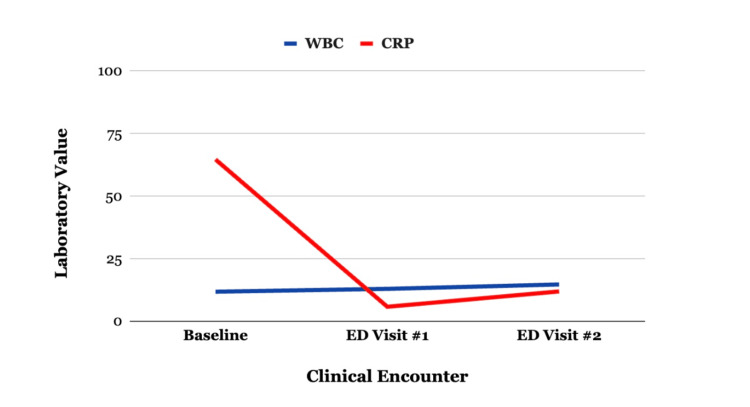
Longitudinal trends in systemic inflammatory markers during the acute illness WBC: white blood cell count; CRP: C-reactive protein; ED: emergency department Line graph demonstrating serial measurements of WBC and CRP from the historical baseline through emergency department visits (ED visit #1 and ED visit #2) during the acute phase of illness

Two days later, he returned to the emergency department with persistent vomiting and worsening rash. Blood pressure reached 143/95 mmHg, representing stage II hypertension for age, although he remained clinically well-appearing without signs of hypertensive emergency. Inflammatory markers were elevated, findings consistent with an ongoing systemic inflammatory process associated with IgAV. Repeat urinalysis remained negative for hematuria and proteinuria; two nonspecific urinary casts were reported, although the laboratory report did not specify the cast morphology, and they were not considered diagnostic of glomerular disease. Laboratory investigations and vital signs across encounters are summarized in Table 1, longitudinal trends in inflammatory markers are illustrated in Figure 2, and the chronological progression of the patient’s illness is shown in Figure 1.

**Table 1:** Comparison of serial laboratory findings and clinical vitals ED: emergency department; WBC: white blood cell count; CRP: C-reactive protein; UA: urinalysis Notes: ↑ indicates a value above the reference range. * Value obtained prior to the acute illness baseline.  ** Pediatric hypertension defined according to the 2017 American Academy of Pediatrics Clinical Practice Guideline for Screening and Management of High Blood Pressure in Children and Adolescents. † Two urinary casts were reported on urinalysis; however, the laboratory report did not specify cast morphology. Hematuria and proteinuria were absent, and the finding was not considered diagnostic of glomerular disease Serial laboratory investigations and vital signs were obtained across multiple clinical encounters, including the historical baseline and emergency department visits during the acute illness.

Parameter	Reference range	Historical baseline (10/14/25)	ED visit #1 (11/27/25)	ED visit #2 (11/29/25)
WBC (10^3^/µL)	4.50-13.50	11.83	12.97	14.72 (↑)
Platelets (10^3^/µL)	150-450	326	405	539 (↑)
CRP (mg/L)	<10.00	64.70 (↑)*	5.81	11.94 (↑)
Creatinine (mg/dL)	0.30-0.70	0.53	0.67	0.50
Blood pressure	<120/80**	Not obtained	126/85 mmHg	143/95 (↑)
Urinalysis protein	Negative	Not obtained	Negative	Negative
Urinalysis blood	Negative	Not obtained	Negative	Negative
Urinalysis casts (number reported)	-	Not obtained	0	2†
Rapid strep	-	Not obtained	Negative	Positive

Over the following several days, the patient experienced continued clinical deterioration with abdominal pain preceding emesis, inability to tolerate oral intake, marked edema of the hands and feet, and new scrotal swelling. He was subsequently admitted for inpatient management.

During hospitalization for approximately two weeks, the patient developed hematochezia with worsening abdominal pain, prompting gastroenterology consultation and endoscopic evaluation that demonstrated diffuse gastrointestinal inflammation and mucosal ulceration. Blood pressure control required initiation of amlodipine 2 mg daily and lisinopril 2.5 mg nightly, with outpatient titration to 10 mg daily. Gastrointestinal mucosal protection included lansoprazole 30 mg daily and sucralfate 0.49 g three times daily. Systemic corticosteroid therapy was administered during hospitalization, followed by a structured oral prednisolone taper beginning at 20 mg daily and gradually decreasing over several weeks. Despite significant systemic disease activity, serial urinalyses during hospitalization remained negative for hematuria and proteinuria.

After inpatient care, gastrointestinal bleeding resolved, and oral intake improved. Blood pressure stabilized on antihypertensive therapy, and the patient was discharged with nephrology and gastroenterology follow-up and plans for serial urinalysis and blood pressure monitoring.

**Figure 3:**
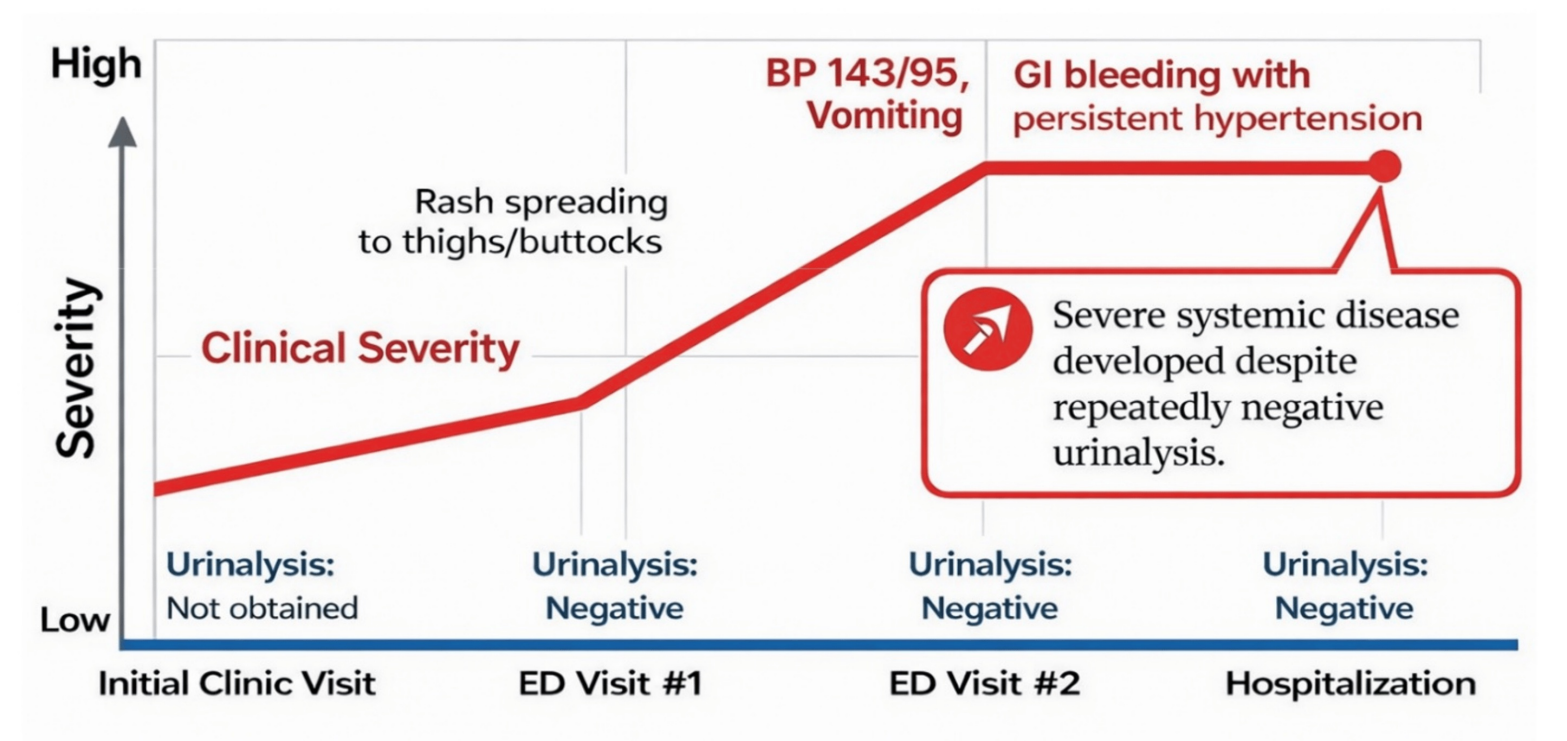
Temporal discordance between disease severity and urinalysis findings BP: blood pressure; GI: gastrointestinal This conceptual diagram illustrates the progression of systemic disease severity during the patient’s acute illness despite repeatedly negative urinalysis results. Key clinical events, including rash progression, vomiting with hypertension, and gastrointestinal bleeding, occurred in the absence of hematuria or proteinuria

At the first outpatient follow-up visit, symptoms were improving, and blood pressure remained controlled on antihypertensive therapy. Ongoing blood pressure and urinalysis surveillance were arranged to monitor for delayed renal involvement. The discordance between progressive systemic disease severity and persistently negative urinalysis findings is conceptually illustrated in Figure 3.

Subsequent outpatient nephrology follow-up demonstrated evolving renal involvement with improvement over time. The urine protein-to-creatinine ratio decreased from 1.7 to 0.8, with persistent but improving microscopic hematuria. Serum creatinine remained normal at 0.39 mg/dL, consistent with stage 1 chronic kidney disease secondary to IgAV. Blood pressure improved with continued therapy using amlodipine and lisinopril.

Endoscopic biopsies performed during hospitalization demonstrated focal active duodenitis and mild colitis, consistent with inflammatory gastrointestinal involvement associated with IgAV. At gastroenterology follow-up several weeks later, abdominal pain and gastrointestinal bleeding had resolved, and the patient had returned to normal activities, with gradual tapering of corticosteroid therapy.

## Discussion

Pediatric IgAV can present with severe systemic manifestations, including gastrointestinal involvement and hypertension, even when initial urinalysis findings are normal. IgAV is the most common systemic vasculitis of childhood, typically presenting between seven and eight years of age and characterized by immune-complex deposition within small vessels leading to the classic tetrad of palpable purpura, arthralgia, abdominal pain, and renal involvement [4]. Gastrointestinal involvement in pediatric IgAV ranges from mild abdominal pain to significant bleeding requiring hospitalization [3]. Recent observational studies have explored the association between gastrointestinal manifestations and the subsequent development of renal complications [6]. Elevated inflammatory markers and reactive thrombocytosis are commonly observed in IgAV and reflect the systemic inflammatory response associated with immune complex-mediated small-vessel vasculitis [7,8]. These findings highlight the systemic inflammatory nature of IgAV and provide important context for atypical presentations in which severe systemic manifestations may occur despite initially normal urinalysis.

Renal disease remains the most clinically important complication of IgAV and is the primary determinant of long-term morbidity [1]. Renal involvement occurs in approximately 20-50% of pediatric patients with IgAV, with the majority of cases developing within the first three months after disease onset [4,7]. Compared with children, adult IgA V is more frequently associated with persistent renal disease and a higher risk of progression to chronic kidney disease [1]. Because renal inflammation may evolve, an initially normal urinalysis does not exclude the later development of nephritis, supporting current recommendations for prolonged surveillance with serial urinalysis during follow-up. This temporal pattern of renal involvement helps explain why early urinalysis may appear reassuring even during periods of significant systemic disease activity. The elevated inflammatory markers and reactive thrombocytosis observed in this patient are consistent with the systemic inflammatory activity characteristic of active IgAV. Although elevated CRP and platelet counts have been associated with internal organ involvement, they are not reliable standalone predictors of renal complications, which remain the principal determinant of long-term prognosis [2,4,6].

The pathogenesis of IgAV is closely related to the “four-hit” hypothesis originally described in IgA nephropathy. In this model, mucosal immune dysregulation leads to the production of galactose-deficient IgA1 (Gd-IgA1), which subsequently forms circulating immune complexes with anti-glycan antibodies. These complexes deposit within small-vessel walls and trigger complement activation, neutrophil recruitment, and leukocytoclastic inflammation in affected tissues, including the skin, gastrointestinal tract, and kidneys. This immune-complex-mediated vascular injury explains the multisystem manifestations observed in IgAV [4,9].

Hypertension in IgAV is most commonly associated with renal involvement and glomerular inflammation. Immune complex deposition within the glomerulus activates mesangial cells and promotes local production of inflammatory mediators and components of the renin-angiotensin-aldosterone system, contributing to sodium retention, vasoconstriction, and elevations in systemic blood pressure. Although hypertension typically develops in the setting of established nephritis, epidemiologic studies demonstrate that patients with IgAV are at increased long-term risk of hypertension compared with controls, reflecting the impact of renal microvascular inflammation on systemic vascular regulation [4,9].

Corticosteroids are frequently used for symptom control in moderate to severe gastrointestinal manifestations of IgAV; however, current evidence indicates that early corticosteroid therapy does not prevent the development of IgAV nephritis in patients without renal involvement. Randomized trials and systematic reviews have demonstrated no significant reduction in persistent nephritis with early glucocorticoid treatment [8]. Consequently, contemporary clinical guidelines recommend against prophylactic corticosteroid use for prevention of renal disease, emphasizing instead the importance of serial urinalysis to detect hematuria or proteinuria as early indicators of renal involvement [7]. Corticosteroids may still shorten the duration of abdominal symptoms and other systemic manifestations during the acute phase of illness [9]. For refractory cases, alternative therapies such as dapsone or intravenous immunoglobulin have been described [10,11], and additional systemic treatment strategies continue to be explored [12].

However, this case illustrates that significant systemic disease and hypertension may occur despite initially normal urinalysis findings. Published literature describes hypertension in IgAV most commonly in association with established nephritis, typically occurring after the appearance of hematuria or proteinuria during the disease course [13].

In contrast, our patient developed sustained hypertension during the acute phase despite repeatedly normal urinalysis. Although this observation does not establish hypertension as an early marker of renal inflammation, it demonstrates that clinically significant disease activity may occur even when standard renal screening appears reassuring. Accordingly, serial blood pressure monitoring may provide additional clinical information alongside urinalysis during disease surveillance rather than being reserved only for cases with confirmed nephritis [14,15].

## Conclusions

This case highlights a potential diagnostic challenge in pediatric IgAV: severe systemic involvement, including gastrointestinal hemorrhage and secondary hypertension, can occur even when traditional renal screening tools such as urinalysis remain repeatedly normal. Clinicians should therefore avoid relying solely on the absence of hematuria or proteinuria to gauge disease severity. Based on this single observation, we hypothesize that serial blood pressure monitoring may serve as a useful complementary surveillance measure alongside urinalysis during both the acute and follow-up phases of disease. Further investigation is needed to determine whether early hypertension may reflect evolving systemic or renal inflammation in IgAV. Prolonged, multimodal monitoring may therefore help ensure that evolving systemic complications are identified and managed promptly, even when initial laboratory findings appear reassuring.
